# Outcomes and complications of postoperative seroma cavities following soft-tissue sarcoma resection

**DOI:** 10.3389/fonc.2024.1250069

**Published:** 2024-01-31

**Authors:** Logan M. Andryk, John C. Neilson, Adam N. Wooldridge, Donald A. Hackbarth, Meena Bedi, Keith E. Baynes, John A. LoGiudice, Sonia M. Slusarczyk, David M. King

**Affiliations:** ^1^ Department of Orthopaedic Surgery, Medical College of Wisconsin, Milwaukee, WI, United States; ^2^ Department of Radiation Oncology, Medical College of Wisconsin, Milwaukee, WI, United States; ^3^ Department of Radiology, Medical College of Wisconsin, Milwaukee, WI, United States; ^4^ Department of Plastic Surgery, Medical College of Wisconsin, Milwaukee, WI, United States

**Keywords:** seroma, sarcoma, infections, soft tissue tumor, fluid collection

## Abstract

**Introduction:**

Seroma development is a known complication following extremity and trunk soft-tissue sarcoma (STS) resection. The purpose of this study is to evaluate and characterize seroma outcomes and the development of associated complications.

**Methods:**

A retrospective review of 123 patients who developed postoperative seromas following STS resection at a single institution was performed. Various patient and surgical factors were analyzed to determine their effect on overall seroma outcomes.

**Results:**

77/123 seromas (62.6%) were uncomplicated, 30/123 (24.4%) developed infection, and 16/123 (13.0%) were symptomatic and required aspiration or drainage for symptom relief at an average of 12.2 months postoperatively. 65/123 (52.8%) seromas resolved spontaneously at an average time of 12.41 months. Seromas in the lower extremity (p=0.028), surgical resection volume >864 cm3, (p=<0.001) and initial seroma volume >42 cm3 (p=<0.001) increased the likelihood of infection. 90% of infected seromas developed the infection within the first three months following initial resection. No seromas which were aspirated or drained ultimately developed an infection following these procedures, though 50% recurred.

**Discussion:**

Most seromas following STS resection are uncomplicated and do not require intervention, though a large resection cavity >864 cm3 and a large seroma volume >42 cm3 are risk factors for complications.

## Introduction

Soft-tissue sarcomas (STS) are rare mesenchymal tumors with >75 subtypes, representing approximately 1% of all malignancies (1–3). The American Cancer Society estimates that 13,190 individuals will be diagnosed with STS in 2022, while 5,130 individuals will pass away from the disease in this same year (4). Wide local resection with or without radiation or chemotherapy is the standard of treatment for STS (5, 6). Surgical resection is performed with negative margin to limit the risk of local recurrence (7), which is reported to occur in approximately 10-15% of patients with STS (8, 9). While five-year survival rates for isolated STS are typically around 60-65%, studies have shown that median length of survival after development of pulmonary metastases is approximately one year (10–12). Therefore, adequate treatment of the primary tumor is imperative before development of metastatic disease.

A notable postoperative complication following STS resection is the development of seromas, which are fluid-filled pockets that develop in the dead-space site where tissue was removed during tumor removal surgery. Seromas are reported to occur in approximately 8-40% of patients following STS resection and have been associated with prior radiation therapy and significant post-resection dead space (13–17). A seroma may be identified clinically or incidentally on follow-up imaging as a well-defined fluid cavity with a thin capsule and surrounding tissue edema (14, 15) which is hypointense on T1 MRI imaging, hyperintense on T2 MRI imaging, and non-enhancing with contrast administration. Prevention strategies have previously been studied and include the use of closed-suction drains, drain removal when output is below 20-50 cc over a 24-hour period, vessel ligation, and postoperative surgical site immobilization (18). Once seromas do develop, their expected clinical course is not well established. While many seromas remain asymptomatic and resolve over time, some can cause discomfort, limit motion, or predispose patients to infection. Given that their natural progression is relatively unknown, they are managed in numerous ways and there is not yet a consensus regarding the appropriate management.

The purpose of this study is to evaluate our historical experience with seromas to further understand their natural history along with the incidence of associated complications, secondary procedures, and factors which may impact overall seroma outcomes. Additionally, we would like to identify risk factors for seroma complications and develop strategies to mitigate them.

## Materials and methods

Following institutional review board approval, a retrospective review was performed for 426 patients who underwent resection of localized STS of the extremity and trunk at our institution from April 2005-September 2021. Inclusion criteria were radiologically or clinically diagnosed seromas and a minimum of two years of radiologic follow-up unless seroma resolution or procedural intervention occurred before this time. If a patient had multiple separately identifiable seromas within their sarcoma resection site, only the largest seroma was followed for the purposes of this study. Subjects were excluded if they had a seroma which was identified preoperatively, there was less than two years of follow-up without resolution or intervention, or if radiographic findings were more suggestive of a hematoma or other diagnosis. Retroperitoneal or abdominal sarcomas were also not considered for the purposes of this study. 123 patients (28.9%) ultimately met inclusion criteria. Primary outcomes included seromas which were uncomplicated and did not require intervention, seromas which developed infection, and symptomatic seromas requiring aspiration or drainage. These outcomes were then evaluated along with various patient and surgical characteristics such as resection volume, drain use, medical comorbidities, and smoking status to determine whether certain patient characteristics are associated with adverse outcomes. Secondarily, seromas which spontaneously resolved were analyzed for time to resolution based on their initial volume. All data was obtained through a retrospective review of electronic medical records of patients meeting this inclusion criteria.

112 seromas were identified on postoperative surveillance imaging, while 11 were diagnosed clinically without specific imaging because physical exam revealed clearly identifiable fluid pockets in the resection cavity, and further imaging was not deemed necessary for a diagnosis. Once identified, seromas were tracked both clinically and radiologically at standard follow-up visits to characterize their natural progression. Specific interventions were not routinely performed unless patients developed significant symptoms specific to their seroma. Post-resection, surgical drain placement helped with prevention of seroma formation, and drain removal was generally attempted when the volume output decreased to less than 20-30mL over a 24-hour period, consistent with clinical consensus (19, 20).

For the purposes of this study and in accordance with previous literature, seroma volume and resection volume were calculated using radiologic measurements of length in the anteroposterior, transverse, and craniocaudal planes. Resection volume was obtained using the gross pathology measurements for the resected specimen. If a seroma spontaneously resolved, time to resolution was calculated from the time of initial identification to the time where a seroma was no longer identified on follow-up imaging. Our institution followed standard National Comprehensive Cancer Network guidelines for STS surveillance. In general, the first post-surgical MRI of the local site was obtained 3-6 months following surgical resection. Subsequent imaging for high-risk patients occurred at 3-4 month intervals for the first 2 years followed by every 6 months from year 2-5, and yearly thereafter. Patients with lower grade tumors were generally surveilled at 6 month intervals for the first 5 years and yearly thereafter to a 10 year follow-up.

Statistical analysis was performed using two-tailed Chi-Squared testing to evaluate the effect of these individual variables on overall seroma outcome. In order to convert the continuous nature of both the seroma and resection volumes into discrete variables for statistical analysis, specific cut-off points were obtained from receiver operating characteristic curves. Unpaired two-tailed t-testing was also utilized to evaluate how average seroma volumes may be related to different outcomes. The threshold for statistical significance was set at p<0.05.

## Results

The seroma patient population included more female patients, at 69/123 (56.1%). Patients ranged from 20-95 years of age at the time of sarcoma resection surgery (average = 57.31 years). Total follow-up ranged from 1.5 months-15 years (average = 4.86 years). Sarcoma resection occurred in the lower extremity for 99/123 patients, while 19/123 resections occurred in the upper extremity and 5/123 occurred on the chest wall, back, and neck. 55 patients underwent flap reconstruction of the surgical defect including either fasciocutaneous or musculocutaneous components. Many patients additionally received other adjuvant or neoadjuvant therapies in addition to surgical resection, as 107/123 (87.0%) received radiation and 46/123 (37.4%) received systemic chemotherapy. 96 patients underwent a wide resection of their tumor and 27 underwent a wide re-resection of their tumor following a previous non-oncologic resection. Overall resection volume ranged from 9.45 cm^3^-12,587 cm^3^ with an average value of 1,241.91 cm^3^ (for reference, a 12 cm x 10 cm x 10 cm resection has a volume of 1,200 cm^3^). Average resection volume was larger in lower extremity resections (1,462.25 cm^3^) and resections on the trunk (721.74 cm^3^) compared to upper extremity resections (230.71 cm^3^). Further demographic information can be seen in **Table 1**.

**Table 1:** Demographic breakdown of patients with postoperative seromas.

Patient Demographics	
Total Number of Patients	123
Age at Surgery	20-95 years (average = 57.31 years)
Male/Female	54/69
Sarcoma Location	Lower Extremity – 99 (80.5%) Upper Extremity – 19 (15.4%) Chest, Back, or Neck – 5 (4.1%)
Smoking History	55 (44.7%)
Diabetes History	15 (12.2%)
Body Mass Index	16.76-58.53 (average = 30.15)
Resection Volume	9.45 cm^3^-12,587 cm^3^ (average = 1,241.91 cm^3^)
Radiation Therapy	107 (87.0%)
Chemotherapy	46 (37.4%)
Flap Coverage	55 (44.7%)
Intraoperative Drain Placement	102 (82.9%)

**Figure 1:**
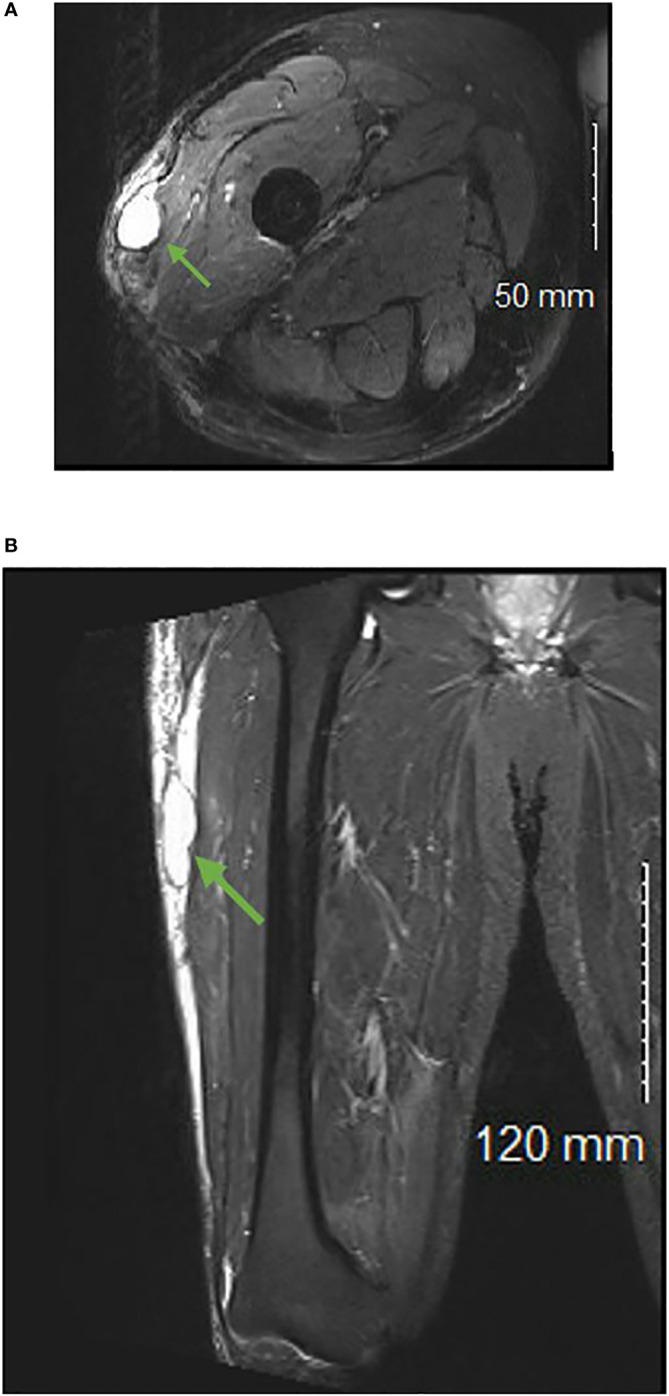
T2 weighted axial **(A)** and coronal **(B)** images of a patient with a postoperative seroma identified in the subcutaneous tissue of the right lateral thigh.

On average, seromas were initially identified 4 months following sarcoma resection (range = 5 days-37 months) and initial volume averaged 140.13 cm^3^ (range = 0.31 cm^3^-1790.1 cm^3^). Representative images of a seroma can be seen in **Figures 1A, B**.

65/123 (52.8%) seromas were found to resolve spontaneously at an average time to resolution of 12.41 months (range = 2-116 months). The average volume of these seromas was 23.15 cm^3^. 29 (23.6%) seromas resolved ≤6 months after initial identification, 46 (37.4%) resolved within one year of identification, and 59 (48.0%) resolved within 2 years. Three seromas resolved between two and four years and three seromas persisted for more than four years before eventually resolving, with one notable seroma being present for 9.7 years before complete resolution. In addition to the six seromas which resolved after a two-year period, 12 (9.8%) other seromas were not intervened upon but were found to be persistent for more than two years and did not resolve at the time of final follow-up. These seromas, though persistent, did not require intervention because of their asymptomatic nature. While nine of these persistent seromas remained at a constant volume or slightly decreased in volume at the most recent follow-up, three persistent seromas increased in volume at final follow-up. The average volume of the seromas which did not require intervention based on their time to resolution can be seen in **Table 2**.

**Table 2:** Average volume of seromas based on time to spontaneous resolution in patients whose seromas were uncomplicated and did not require intervention.

Time to Resolution	Number of Patients	Average Volume (cm^3^)
≤6 months	29	12.11
Between 6 months and 1 year	17	26.39
Between 1 year and 2 years	13	30.57
>2 years	6	51.85
No resolution >2 years	12	288.72

The presence of a seroma was not found to be an independent risk factor for infection, as 30/123 seromas (24.4%) became infected while 50/303 (16.5%) sarcoma resections without a seroma experienced an infection (p=0.059). 26 of the 30 infected seroma patients underwent open surgical debridement, while four patients were managed with aspiration or drainage alongside an antibiotic regimen. 26/30 (86.7%) infected seromas were infected within the first three months following initial surgical resection. One seroma developed infection at four months post-resection following an in-office debridement of necrotic subcutaneous fat. Another seroma developed infection after previously being stable for six months, coinciding with the induction of a new immunotherapy and a notable drop in the patient’s white blood cell count. Two other infections developed between six months and one year postoperatively with no identifiable cause, and no infections developed in persistent seromas more than one year following initial surgical resection. 26/30 (86.7%) patients with an infected seroma had postoperative drain placement, with 19 (73.1%) developing infection after drain removal at a mean length of 1.26 months (range 5 days – 5.5 months). Seven (26.9%) of these patients developed infection while the drain remained present.

Seromas which developed infection after drain placement on average had their drain present for 0.83 months before removal, compared to 0.78 months for noninfected seromas (p=0.699). 26/30 (86.7%) seromas ultimately resolved after the infection was managed, though four seromas recurred and persisted after an incision and drainage procedure. None of these four persistent seromas re-developed infection.

16 seromas (13.0%) were not infected but were otherwise found to be notably symptomatic (often in the form of pain, chronic drainage, or significant swelling), leading to aspiration or surgical drainage at an average time of 12.2 months postoperatively (range = 2 weeks – 89 months). Following this intervention, eight seromas (50%) resolved and did not recur, while eight other seromas recurred in the same cavity. Of the eight seromas which recurred, six recurred at a smaller volume than prior to intervention, while two seromas (12.5% of all aspirated or drained seromas) recurred at a larger volume than prior to intervention. None of these 16 seromas developed infection following aspiration or drainage.

Chi-square analysis was utilized to evaluate the individual impact of various patient factors on seroma infection risk, as seen in **Table 3**. Seromas were statistically more likely to become infected if the initial seroma volume was >42 cm^3^ (35.4% vs. 7.8%, p=<0.001), if the tumor resection volume was >864 cm^3^ (46.8% vs. 10.5%, p=<0.001), or if resection occurred in the lower extremity rather than the upper extremity (29.3% vs. 5.3%, p=0.028).

**Table 3:** Analysis of patient and surgical characteristics which may be associated with seroma infection.

	Total n=123	No Infection (n=93, 75.6%)	Infection (n=30, 24.4%)	p-Value
Sex	Male (n=54)	44	10	0.180
	Female (n=69)	49	20	
Age at Surgery (years)	≤57 (n=62)	48	15	0.878
	>57 (n=61)	45	15	
Smoking History	Yes (n=55)	37	18	0.053
	No (n=68)	56	12	
Diabetes History	Yes (n=15)	10	5	0.389
	No (n=108)	83	25	
Body Mass Index	<30 (n=73)	56	17	0.731
	>30 (n=50)	37	13	
Radiation	Yes (n=107)	80	27	0.573
	No (n=16)	13	3	
Chemotherapy	Yes (n=46)	38	8	0.162
	No (n=77)	55	22	
Resection Volume (cm^3^)	≤864 (n=76)	68	8	**<0.001**
	>864(n=47)	25	22	
Initial Seroma Volume (cm^3^)*	≤42 (n=64)	59	5	**<0.001**
	>42 (n=48)	31	17	
Intraoperative Drain Placement^	Yes (n=102)	76	26	0.303
	No (n=15)	13	2	
Flap Coverage	Yes (n=55)	38	17	0.130
	No (n=68)	55	13	
Resection Location	Upper Extremity (n=19)	18	1	**0.028**
	Lower Extremity (n=99)	70	29	

*Eleven patients experienced seromas which were not officially measured and were diagnosed clinically.

^Intraoperative drain placement was not documented for six patients.

Bold values indicate statistical significance, with a p-value <0.05.

Of the 29 infected seromas in the lower extremity, the thigh was the most commonly affected region (20), while the pelvis (5), and leg (4) were less commonly affected. 9/20 (45%) infected seromas in the thigh were found in the anterior compartment, 6/20 (30%) were found in the medial compartment, and 5/20 (25%) were in the posterior compartment.

There were found to be statistically significant differences in the mean volumes of seromas which did not require intervention and those which did. The mean volume of seromas which were infected (235.31 cm^3^) and aspirated or drained (389.85 cm^3^) were significantly higher than the mean volume of those seromas which were uncomplicated (65.08 cm^3^, p=<0.001, **Table 4**).

**Table 4:** Mean seroma volumes based on overall outcome.

	Mean Volume (cm^3^)	p-Value (Compared to Uncomplicated)
Uncomplicated	65.08	
Infection	235.31	<0.001
Aspiration/Drainage	389.85	<0.001

## Discussion

Seroma development is a notable risk following STS resection given the large potential space created after surgical resection and they have been reported to occur following approximately 8-40% of resections. Risk factors influencing seroma development have been studied relatively thoroughly, particularly following breast surgery (21–24), though the natural history and outcomes of seromas has been minimally studied overall. A few studies have described the imaging features of postoperative seromas to determine an accurate identification method and to secondarily evaluate their natural progression (13–15, 25). For example, one study analyzing magnetic resonance imaging findings of seromas following STS resection (25) found that seroma volume decreased naturally in 66% of cases, while 19% increased in volume postoperatively. These studies have helped improve diagnosis and patient expectations, but none have yet addressed the potential complications associated with seroma development and factors which may influence their overall outcomes. Our study is, to our knowledge, the largest study which evaluates the natural history, outcomes, and complications associated with postoperative seromas following STS resection.

One similar study by El Abiad (26) evaluated seroma history in 48 patients following STS resection, 42 of whom had no specific intervention performed for seroma management. Almost all of these seromas (46/48) were found in the lower extremity. They found that seroma resolution occurred in 20/48 (42%) patients at a mean time of 50 months post-STS resection. A higher resolution rate was found in men, patients with a seroma volume <85 cm^3^, and those who did not undergo radiation therapy. Similarly, our study found that seromas with smaller volumes were more likely to spontaneously resolve, as our mean volume of seromas which spontaneously resolved was 23.15 cm^3^ compared to 235.31 cm^3^ for infected seromas and 389.85 cm^3^ for symptomatic seromas requiring aspiration or drainage (p=<0.001). Our study, however, showed a slightly higher percentage of spontaneous resolution (53%) and our average time to resolution was much shorter at 12.41 months. Of the 65 seromas which did spontaneously resolve in our study, 59 (90.8%) resolved within two years of initial identification.

Although 62.6% of seromas were ultimately uncomplicated, 24.4% developed infection and 13.0% required aspiration or drainage. The risk of infection was found to be related to overall resection and seroma volumes, being statistically more likely with a resection cavity >864 cm^3^ and an initial seroma volume >42 cm^3^. For these large resections, a fasciocutaneous or musculocutaneous flap was often utilized for wound coverage, as this type of reconstruction has been shown to minimize dead space and lower the likelihood of seroma formation (27). At our institution, both local and free flaps were routinely used for coverage depending on the resulting defect. Additionally, a lower extremity seroma was statistically more likely to become infected than an upper extremity seroma; however, we believe the increased likelihood of infection in lower extremity resections is predominantly related to seroma volume and not necessarily anatomic location, as the volume of infected seromas in the lower extremity was notably larger than the average in our cohort. Therefore, we believe the volume of both the resection and the resultant seroma are the most important influencing factors on the development of seroma complications.

Given that these larger seromas are less likely to spontaneously resolve over time and the persistent presence of a large amount of fluid in a body cavity increases the opportunity for infection, we were concerned that persistent seromas may have an increased frequency of late-breaking infections; however, we did not see this clinically, as only four seromas developed an infection further than three months following STS resection. Most infections occurred either shortly after surgical drain removal or while the surgical drain was still present. We believe that it is likely that these seromas which were infected within a short time of drain removal were sub-clinically present and removal of the drain simply unmasked a pre-existing infection which previously had an outlet for drainage. While the surgical drain helps provide this outlet for fluid, it is also important to avoid inducing infection through prolonged drain use, as previous studies have shown the likelihood of infection increases after a range of 3 days to 3 weeks of drain placement (24, 28, 29). Our study found no significant difference in the length of drain placement between infected and noninfected seromas and did not identify drain use as a statistically significant risk factor for the development of infection.

For seromas which were symptomatic enough to warrant aspiration or drainage, much of these symptoms came in the form of painful swelling or chronic drainage, and these seromas were typically the largest volume seromas with an average volume of 389.85 cm^3^. Given that larger oncologic resections and their subsequent seromas have a higher propensity for significant symptoms and infection, it may be clinically appropriate to consider prophylactic aspiration of large seromas >42 cm^3^ to lessen the potential for infection and potentially reduce the need for future procedures. There is some hesitation in intervening on these seromas because of the theoretical possibility of introducing infection; however, the results of this study show that 0/16 patients who underwent aspiration or drainage at an average of 12.2 months following tumor resection ultimately experienced infection. It is notable that 50% of these seromas recurred after intervention, though only two recurrent seromas were larger than their initial size. This study also shows that infection of seromas generally happens within the first three months following tumor resection, and the likelihood of spontaneous infection in a persistent seroma after this timeframe is low. Therefore, prophylactic intervention is most likely to be effective at reducing infection risk within the first three months following tumor resection. The decision to prophylactically intervene on large seromas should be weighed against the patient’s clinical status, though it may be especially important if the patient has other significant risk factors for infection such as advanced age, diabetes mellitus, immunodeficiencies, or a smoking history.

Previous studies have also described adjunct radiation therapy as a risk factor for infection, wound healing complications, seroma development, and seroma persistence. In this study, radiation therapy was found to ultimately not impact overall outcomes, though it is important to note that most patients at our institution undergo radiation therapy alongside surgical resection. The low percentage of patients who did not receive radiation makes it less likely that we would identify a strong relationship between radiation therapy and seroma outcomes if a relationship truly existed.

There are some notable limitations to this study. First, seroma volumes were approximated by multiplying the radiologic lengths in the anteroposterior, transverse, and craniocaudal planes, which leads to slight volume inaccuracies because of the complex three-dimensional shapes of seromas. Second, seroma time to development and time to resolution may not be entirely accurate, as radiologic surveillance was typically performed at standard follow-up visits. The gap between repeat images likely leads to an overestimation of both the time to formation and time to resolution. Third, the volume of seromas was determined at the time of initial diagnosis of the seroma itself. For patients with infections, this volume was often determined prior to the onset of infection; however, some seromas were identified after the onset of infection and the size may have been inflated by the infection itself. Finally, the length of follow-up varied significantly among patients, and seromas which may have otherwise been considered persistent ultimately resolved in patients with extensive postoperative surveillance, such as in the patient whose seroma resolved after 9.7 years. Similarly, seromas which have not yet resolved at the time of this study may ultimately resolve spontaneously with long-term follow-up.

## Conclusions

The volumes of both the initial STS resection and the resultant seroma are important contributing factors to the overall seroma outcome, as tumor resection volume >864 cm^3^ and initial seroma volume >42 cm^3^ were found to statistically increase the likelihood of infection. Most seromas will resolve spontaneously without complications, though some will develop infection or persistent symptoms. Infection is most likely to occur within the first three months postoperatively and seromas present for more than three months are unlikely to spontaneously become infected. Additionally, the risk of subsequent infection following aspiration or drainage is very low.

## Data availability statement

The raw data supporting the conclusions of this article will be made available by the authors, without undue reservation.

## Ethics statement

The studies involving humans were approved by Medical College of Wisconsin Institutional Review Board #5. The studies were conducted in accordance with the local legislation and institutional requirements. Written informed consent for participation was not required from the participants or the participants’ legal guardians/next of kin because this was a retrospective review.

## Author contributions

LA worked on data collection, formal analysis, investigation, statistics, and writing of the manuscript. JN, AW, JL, DH, MB, KB, and DK conceptualized the project, performed investigation and patient care, and worked on writing of the manuscript. SS worked on data collection, investigation, and formal analysis. All authors contributed to the article and approved the submitted version.
